# Comparison of Placenta Previa and Placenta Accreta Spectrum Disorder Following Previous Cesarean Section between Women with a Short and Normal Interpregnancy Interval

**DOI:** 10.1155/2022/8028639

**Published:** 2022-08-03

**Authors:** Uchenna Anthony Umeh, George Uchenna Eleje, Justus Uchenna Onuh, Ogochukwu Theophilus Nwankwo, Ijeoma Victoria Ezeome, Leonard Ogbonna Ajah, Ngozi Regina Dim, Samuel Nnamdi Obi, Chidebe Christian Anikwe, Joseph Ifeanyichukwu Ikechebelu

**Affiliations:** ^1^Department of Obstetrics and Gynecology, College of Medicine, University of Nigeria, Enugu Campus, Enugu, Nigeria; ^2^Effective Care Research Unit, Department of Obstetrics and Gynecology, Faculty of Medicine, Nnamdi Azikiwe University, Awka, Nigeria; ^3^Department of Obstetrics and Gynecology, Nnamdi Azikiwe University Teaching Hospital, Nnewi, Nigeria; ^4^Department of Psychology Medicine, Nnamdi Azikiwe University, Awka, Nigeria; ^5^Department of Radiation Medicine, College of Medicine, University of Nigeria, Enugu Campus, Enugu, Nigeria

## Abstract

**Objectives:**

The aim of this study is to determine the effect of interpregnancy interval (IPI) on the incidence of placenta previa and placenta accreta spectrum disorders in women with a previous cesarean section.

**Methods:**

A prospective cohort three-center study involving parturients who had previous cesarean section was conducted. Participants were included if pregnancy has lasted up to 34 weeks. Parturients with co-existing uterine fibroids, multiple gestations, premature rupture of membranes, and those with prior postcesarean delivery wound infection were excluded. The eligible women recruited were distributed into two groups, namely, short (<18 months) and normal (18–36 months) IPI. The outcome measures were incidences of placenta previa and placenta accreta spectrum disorder and factors associated with the occurrence of placenta previa. A univariate analysis was performed using the chi-square test or Mann–Whitney *U* test, wherever appropriate, to examine the significance of the differences in clinical variables.

**Results:**

A total of 248 women met the inclusion criteria. The incidence of placenta previa by ultrasound was 8.9% and 4.0% for short and normal IPI (odds ratios = 2.32; 95% confidence intervals = 0.78–6.88; *p* = 0.13), respectively. The incidence of placenta accreta spectrum disorder was 1.6% and 0.8% for short and normal IPI (odds ratios = 2.02; 95% confidence intervals = 0.18–22.13; *p* = 0.57), respectively. The only observed significant difference between the clinical variables and placenta previa is the number of cesarean sections (*p* = 0.02) in women with short IPI.

**Conclusion:**

A short interpregnancy interval does not significantly affect the incidence of placenta previa and placenta accreta spectrum disorder following a cesarean section. There is a need for further study with large numbers to corroborate these findings in low- and middle-income settings.

## 1. Introduction

Interpregnancy interval (IPI) is defined as the time lapse between two consecutive pregnancies [1]. It has also been defined as the time between the end of one pregnancy and the beginning of the next pregnancy [2]. Normal IPI is defined as IPI between 18 and 36 months. Short IPI is defined as IPI less than 18 months [3]. However, different studies have used various definitions for short IPI such as less than 3, 6, 9, 12, or 18 months. Long IPI is defined as IPI greater than 36 months [3].

An IPI less than 6 months is most often associated with adverse outcomes. Some studies have reported correlations with adverse outcomes for IPI less than 3 months or IPI less than 18 months [4–6]. Women who have short IPIs of less than 18 months have been linked with an increased risk of adverse perinatal outcomes, including placenta previa [7, 8]. Studies have shown the association between the short IPIs and risk of placenta previa for women who were previously delivered by a cesarean section [8–10].

There is robust literature on the risk factors of placenta previa in Western and Asian countries. However, less attention has been directed towards investigating the effect of short IPI on placenta previa following previous cesarean section [11]. Studies in this regard have shown inconsistent results [8, 11]. Conde-Agudelo et al. [11] conducted a systematic review to explain the causal mechanisms of the effect of IPIs on pregnancy outcomes.

However, there is paucity of data on the relationship between IPI and the risk of placenta previa and placenta accreta spectrum disorder in low-income settings where there are high aversions for cesarean section [8, 12, 13]. It is worthwhile to note that the majority of previous studies were conducted in the western populations [3, 11, 14]. The findings of this study will aid in highlighting the potential of the application of birth spacing as an intervention for ameliorating maternal and perinatal morbidity and mortality. This information will enlighten healthcare providers, particularly midwives and obstetricians on the possible risk of placenta previa in women with previous cesarean section with short IPI. Findings will also enrich the available data on this area of study and serve as a reference in future research for other researchers. The aim of this study is to determine and compare the incidence of placenta previa and the placenta accreta spectrum disorder following a previous cesarean section between women with short IPI and those with normal IPI.

## 2. Materials and Methods

### 2.1. Study Site

The study sites were University of Nigeria Teaching Hospital (UNTH), Ituku/Ozalla Enugu, Nigeria; Enugu State University Teaching Hospital (ESUTTH), Parklane, Enugu, Nigeria, and Mother of Christ Specialist Hospital (MCSH), Enugu, Nigeria.

### 2.2. Study Population

The study population were consenting pregnant women with previous cesarean section attending antenatal clinics.

### 2.3. Study Design

This was a prospective cohort study.

### 2.4. Inclusion Criteria

Parturients who had a previous cesarean section and were currently pregnant at 34 or more weeks of gestation were included in the study.

### 2.5. Exclusion Criteria

Parturients with co-existing uterine fibroids, multiple gestations, premature rupture of membranes (PROM), and a history of previous postcesarean delivery wound infection were excluded from the study.

### 2.6. Sample Size

We estimated that a sample size (with continuity correction) of 214 with a 1 : 1 case to control ratio (107 cases and 107 controls) would allow us to accept a two-tailed alpha error of 0.05 and beta error (type II error) of 0.10 at 90% power using the prevalence of placenta previa of 26.7% [15] for women with short IPI following previous cesarean section and an expected decrease of 10.0% in the controls with normal IPI. However, we recruited 139 participants in each of the case and control groups to account for possible loss to follow-up.

The participants were placed in two groups as cases: Group A (short IPI) and controls: Group B (normal IPI). All eligible women attending antenatal clinics at UNTH, ESUTTH, and MCSH were consecutively recruited at 34 weeks gestation into the 2 groups.

For the purpose of this study, short IPI was defined as women with an IPI of less than 18 months [3], while normal IPI was defined as an IPI of 18–36 months [3]. The cutoff of 36 months was chosen to limit the cofounding effect of subfertility and likely change in partner. Written informed consent was obtained from all the participants in the study.

### 2.7. Study Procedure

Data were collected using a predesigned proforma for the study. Information obtained included sociodemographic characteristics (maternal age, marital status, religion, and educational status) and clinical variables (parity, reproductive history, past obstetric history including last confinement and outcome). The last menstrual period and pregnancy intention (intended, mistimed, or unwanted) were also documented. Clinical examinations were carried out and maternal height, weight, and blood pressure were recorded. An ultrasound scan was done at 34 weeks gestation to determine the placenta location, and the pregnancy outcome was documented to confirm or exclude placenta previa at delivery. Also, the mode of delivery and number of previous cesarean sections were noted.

Three trained research assistants were engaged in each of the three study sites. The three assistants were two resident doctors in Obstetrics and Gynecology and an experienced radiologist. The resident doctors assisted in the collection of data using the predesigned proforma while the radiologist performed the obstetric scan on the participants.

A Toshiba model ultrasound machine with a 3.5 MHz curvilinear transducer or a 5.0 MHz transvaginal transducer was used to scan the participants and measurements were taken in freeze mode by a single observer with experience in obstetric sonography. Ultrasound assessment was performed using a transabdominal or transvaginal probe as indicated. A transabdominal longitudinal scan of the placenta was performed with the parturients in the supine position and with a full bladder. Subsequently, transvaginal scan of the placenta was performed on the parturients after emptying the bladder. The relationship between the placenta and internal cervical os was assessed. If the internal cervical os was visualized and no placental tissue overlies it, placenta previa was excluded. Also, the following findings excluded placenta previa: direct apposition of the presenting part of the fetus and the cervix without space for interposed tissue; the presence of amniotic fluid between the presenting part of the fetus and the cervix, without the presence of placental tissue; and a distance of greater than 2 cm between the inferior aspect of the placenta and the internal cervical os on direct visualization [16]. All participants were followed up till delivery to ascertain the actual placenta position and mode of delivery and any maternal or fetal morbidity or mortality.

### 2.8. Outcome Measures

The outcome measures include the incidence of placenta previa, placenta accreta spectrum disorder, and factors associated with occurrence of placenta previa.

### 2.9. Statistical Analysis

Data were coded, entered, and analyzed using SPSS version 20. Descriptive statistics which included frequency and percentages were used to summarize categorical variables such as the incidence of placenta previa. The associations between the clinical variables were determined using the chi-square test or Mann–Whitney *U* test wherever appropriate. The odds ratios (ORs) with 95% confidence intervals (CIs) were used to quantify the incidences and a statistical significance was accepted at *p* value less than 0.05. The results are presented in tables and figures.

### 2.10. Ethical Consideration

The approval for this study was obtained from the Ethics Committee of University of Nigeria Teaching Hospital (NHIREC/05/01/2008B-FWA0000245B-IRB00002323, 11^th^ February 2019). The proposed study commenced after approval by the Institute of Maternal and Child Health. There was individual counseling for each participant recruited for the study and informed written consent was obtained.

## 3. Results

**Figure 1:**
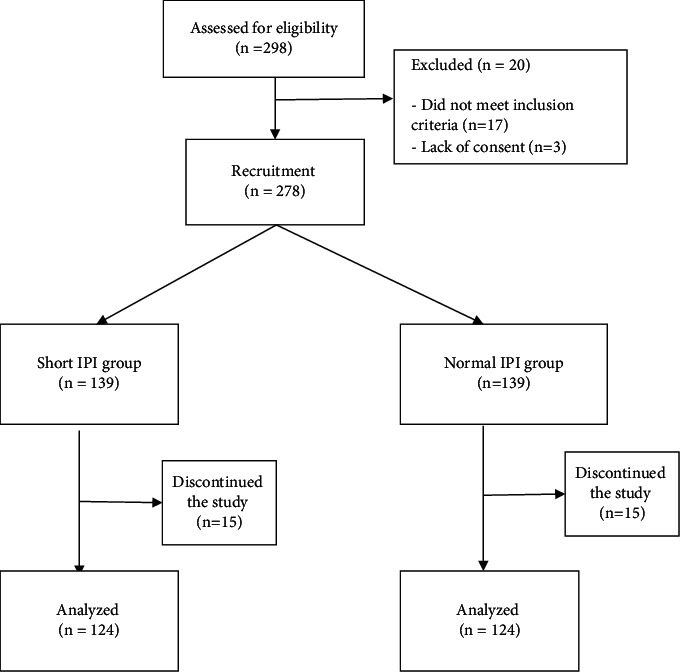
Flowchart of the study participants.

A total of 298 women were assessed for eligibility in the study. Two hundred and seventy-eight women met the inclusion criteria and were recruited, while 20 women were excluded from the study. Subsequently, 30 women were lost to follow-up and 124 were followed-up till delivery in each group. The flowchart of the participants is shown in Figure 1. Two hundred and forty-eight women were analyzed, 124 participants for each group. The majority of the participants in the two groups were between the ages of 20 and 34 years, with a mean age of 33.19 ± 4.54 years for group A and 34.06 ± 3.88 years for group B. None of the participants were below 20 years of age. The baseline sociodemographic characteristics of the participants were similar in the two groups as shown in Table 1.

Table 2 shows the clinical characteristics of the study participants. The participants have similar clinical characteristics with respect to parity, number of cesarean sections, mean gestational age at booking, mean systolic blood pressure, and body mass index. However, the mean diastolic blood pressure and pregnancy intention showed a statistical difference between the two groups.

Table 3 shows the comparison of the incidence of placenta previa and placenta accreta spectrum disorder between the two groups. This was similar for the two groups. In Group A, 11 (8.9%) women had placenta previa by ultrasound evaluation while 12 (9.7%) women had placenta previa by inspection. In Group B, 5 (4.0%) women had placenta previa by ultrasound evaluation while 6 (4.8%) women had placenta previa by inspection. Furthermore, in Group A, 2 (1.6%) women had placenta accreta spectrum disorder while 1 (0.8%) woman had it in Group B. The observed differences were not statistically significant (*p* > 0.05).

Table 4 shows the association between sociodemographic factors and the presence of placenta previa using ultrasound in women with a short interpregnancy interval. There was no significant association between demographic variables of age, marital status, educational status, and employment status with placenta previa in women who have short IPI.

Table 5 shows the association between clinical factors and the presence of placenta previa using ultrasound in women with short IPI. There was no observed difference between clinical variables and placenta previa in women with short interpregnancy intervals except for the number of cesarean sections (*p*=0.02). The median for the number of cesarean sections in the 2 groups was 1 and 2, respectively.

All the participants with confirmed placenta previa had cesarean delivery, with cesarean hysterectomy occurring in 7 (5.6%) women with short IPI and in 4 (3.2%) women with normal IPI. Postpartum hemorrhage occurred in 43 (37.4%) women with short IPI and in 32 (25.8%) women with normal IPI. Balloon tamponade was employed in 6 (4.8%) women with short IPI and in 4 (3.2%) women with normal IPI. There was no maternal death in either group.

## 4. Discussion

In this study, the incidence of placenta previa by ultrasound in women with short IPI was 8.9% and by inspection during delivery was 9.7%. Similarly, the prevalence of placenta previa by ultrasound in women with normal IPI was 4.0% and by inspection during delivery was 4.8%. These figures were higher than the previously reported values in the study environment. For example, Iyoke et al. and Ikechebelu et al. reported the prevalence of 3.4% (in 2014) and 1.65% (in 2007) in Enugu, Nigeria, and Nnewi, Nigeria, respectively [17, 18]. However, the incidence rate reported in this study was consistent with that of other studies [14, 19]. For example, Hsieh et al. found a similar identified magnitude of risk for placenta previa (OR: 4.2; 95% CI = 3.0–6.0), after a short and long interval outcome following a cesarean section [19]. The reason for the similarities in findings of these studies with the present study could be explained by the congruence of methodologies used in both studies. Both studies recruited pregnant women in their third trimesters and in the population with previous cesarean sections. The plausible explanation for the differences between the findings of the study and the previous works could be due to differences in sampled population, period of the study, and the method of determining the presence of placenta previa.

With regards to the relationship between IPI and the prevalence of placenta previa and placenta accreta spectrum disorder, this study did not find any significant difference between those with short and normal IPI. The literature on the relationship between IPIs as a risk factor for placenta previa and placenta accreta spectrum disorder was sparse. It appears that the available literature was inconsistent with regard to the relationship between IPI and the prevalence of placenta previa after cesarean section. However, while some studies reported a significant association [19, 20], others found no relationship [15, 21]. The results of these studies suggest that a short IPI may not increase the risk of developing placenta accreta spectrum disorder. The reasons for our findings were not very clear although it could be that the risk of placenta previa and placenta accreta spectrum disorder were similar irrespective of the IPI or that the higher prevalence of placenta previa or placenta accreta spectrum disorder in people with short IPI may be explained by other factors other than the interval between the pregnancies.

With respect to the sociodemographic factors, there was no significant association between demographic variables of age, marital status, educational status, and employment status with placenta previa in women who had short IPI. Additionally, there was no observed difference between clinical variables and placenta previa in women with short IPI except for the number of cesarean sections.

Placenta previa occurs more in women with a previous history of cesarean section and the chance of placenta previa also increases with successive increases in the number of cesarean sections as occurred in our study. This finding was similar to a previous study by Downes et al. [22]. Although there were no significant differences in the occurrence of placenta previa in the two groups, the number of exposures was more with short IPI. Therefore, interventions are needed to address suboptimal birth spacing in this population. Second, women with a first birth at an age of 30 years or older are more likely to experience short IPIs than those initiating childbearing earlier, suggesting that closer birth spacing could be a response to late initiation of childbearing. This premise was supported by the findings of the American College of Obstetricians and Gynecologists (ACOG) in 2009 [23]. ACOG revealed that among pregnancies that had normal IPIs in women initiating childbearing after age 30 years, nearly three out of four were intended pregnancies [23]. Thus, they appear to have a greater number of participants with short IPIs [23].

The choice of an 18-month cutoff to define a short IPI in this analysis was based on the indicator used in Healthy People 2020 [13]. While some authorities suggest that IPI less than 18 months was associated with increased risk, it is important to note that even within this 18-month window, the level of risk decreases as interval length increases [13, 24–27].

All the participants with a confirmed placenta previa had a cesarean section, with cesarean hysterectomy occurring in 7 (5.6%) women with short IPI and in 4 (3.2%) women with normal IPI. Postpartum hemorrhage occurred in 43 (37.4%) women with short IPI and in 32 (25.8%) women with normal IPI. Balloon tamponade was employed in 6 (4.8%) women with short IPI and in 4 (3.2%) women with normal IPI. There was a high number of participants that had postpartum hemorrhage. The findings of this work are similar to a previous work by Wang et al. that analyzed relevant factors for massive postpartum hemorrhage in women with placenta accreta spectrum in order to improve the ability to identify those at risk for intraoperative bleeding and improve outcomes [28]. Wang et al. concluded that the presence of cervical blood sinus, interruption or disappearance of bladder line, the disappearance of the postplacental clear space and abnormal subplacental vascularity are independent risk factors for massive hemorrhage during placenta accreta spectrum disorder management [28]. In another study, Gulino et al. studied the efficacy of using precesarean delivery temporary occlusion of internal iliac arteries with balloon catheters in case of placenta accreta spectrum disorder in terms of maternal and neonatal outcomes and to test the accuracy of ultrasound and magnetic resonance imaging for prenatal diagnosis. The authors concluded that temporal, perioperative, and prophylactic positioning of balloon vascular catheters is an active method for managing severe hemorrhage caused by placenta accreta spectrum disorder as it reduced intraoperative blood loss, lessened perioperative hemostatic measures, intraoperative red cell transfusions, and reduced hysterectomies [29]. Similarly, Hong et al. did a clinical evaluation of the efficacy of IIA balloon occlusion during cesarean sections in patients with a diagnosis of placenta accreta spectrum disorders [30]. Although the complications related to occlusion of IIA balloon did not occur, Hong et al. revealed that more than 40% participants that received balloon occlusion underwent hysterectomy because of uncontrollable postpartum bleeding [30].

The strength of our study was its prospective design since data were collected prospectively. The two groups were similar in clinical characteristics concerning parity, a number of cesarean sections, mean gestational age at booking, mean systolic blood pressure and body mass index. We acknowledge certain limitations of our study, in particular, the study population did not represent those with a phobia for repeat cesarean sections who may have gone to deliver at a health center or maternity home [31]. We did not identify matched control subjects according to the location of the placenta and we did not evaluate whether the cesarean sections were performed electively or during labor [32]. There is a need to evaluate these in future studies. Additionally, transvaginal ultrasound was not performed in all cases because some participants refused consent for TVS because they thought that passing the probe transvaginally would predispose them to vaginal bleeding. In this study, the authors performed the ultrasound so late in pregnancy to evaluate placenta previa because most of the women booked late in the second trimester. According to the *Royal College of Obstetricians and Gynecologists* (*RCOG*) Green-top guideline on placenta previa and placenta accreta, it was suggested that early ultrasound should be performed between 18 + 6 and 21 + 6 weeks of gestation [33].

## 5. Conclusions

Short interpregnancy interval appears not to significantly affect the incidence of placenta previa and placenta accreta spectrum disorder following a cesarean section. However, placenta previa and placenta accreta spectrum disorder occur more in women with a previous history of cesarean section and the chance of placenta previa also increases with successive increase in the number of cesarean sections. Health care providers should be trained in effective health education and counseling skills in order to impart knowledge to women on proper birth spacing and its benefits. Further research to re-evaluate the evidence-based findings of negative health consequences of short interpregnancy intervals among reproductive age women is recommended. There is a need for further study to corroborate our findings with that of others from low- and middle-income settings.

## Figures and Tables

**Table 1:** Sociodemographic characteristics of the study participants.

Variables	Normal IPI (*n* = 124)	Short IPI (*n* = 124)	Test stat	df	*p*-value
Mean age (SD)	34.06 (3.88)	33.19 (4.54)	*t* = −1.63	246	0.11

*Age group (years)*			χ^2^ = 3.19	2	0.07
Less 20	0 (0.0%)	0 (0.0%)			
20–34	61 (49.2%)	75 (60.5%)			
>34	63 (50.8%)	49 (39.5%)			

*Marital status*			χ^2^ = 1.35	2	0.51
Single	0 (0.0%)	0 (0.0%)			
Married	123 (99.2%)	121 (97.6%)			
Divorced	0 (0.0%)	1 (0.8%)			
Widowed	1(0.8%)	2 (1.6%)			

*Educational status*			χ^2^ = 2.78	3	0.43
No formal	2 (1.6%)	2 (1.6%)			
Primary	2 (1.6%)	4 (3.2%)			
Secondary	43 (34.7%)	32 (25.8%)			
Tertiary	77 (62.1%)	86 (69.4%)			

*Employment status*			χ^2^ = 3.19	1	0.07
Employed	117 (94.4%)	109 (87.9%)			
Unemployed	7 (5.6%)	15 (12.1%)			

Religion			χ^2^ = 1.02	1	0.31
Christian	123 (99.2%)	121 (97.6%)			
Others	1 (0.8%)	3 (2.4%)			

NB: IPI = interpregnancy interval, SD = standard deviation, df = degree of freedom.

**Table 2:** Clinical characteristics of the study participants.

Variables	Normal IPI (*n* = 124)	Short IPI (*n* = 124)	Test stat	*p*-value
Parity			*U* = 7633.00	0.20
**Mean (SD)**	2.19 (1.18)	2.13 (0.10)		
**95% CI**	1.98–2.40	1.95–2.30		
**Median**	2.00	2.00		
**Min-Max**	1.00–7.00	1.00–5.00		

Number of C/S			*U* = 7410.50	0.57
**Mean (SD)**	1.48 (0.73)	1.52 (0.72)		
**95% CI**	1.35–1.61	1.39–1.65		
**Median**	1.00	1.00		
**Min-Max**	1.00–4.00	1.00–4.00		

Mean gestational age at booking **(**weeks)			*U* = 6854.50	0.14
**Mean (SD)**	18.12 (5.99)	19.19 (5.77)		
**95% CI**	17.06–19.19	18.16–20.21		
**Median**	17.50	18.00		
**Min-Max**	7.00–31.00	7.00–32.00		

Mean systolic BP (mmHg)			*U* = 6939.50	0.17
**Mean (SD)**	106.21 (13.10)	108.4 (11.97)		
**95% CI**	103.98–108.54	106.30–110.60		
**Median**	110.00	110.00		
**Min-Max**	80.00–140.00	80.00–140.00		

Mean diastolic BP (mmHg)			*U* = 6194.50	0.005
**Mean (SD)**	64.52 (9.89)	67.30 (7.82)		
**95% CI**	62.76–66.27	65.91–68.69		
**Median**	60.00	70.00		
**Min-Max**	50.00–95.00	50.00–90.00		

Body mass index			χ^2^ = 1.16	0.76
**Underweight**	0 (0.0%)	1 (0.8%)		
**Normal**	4 (3.2%)	3 (2.4%)		
**Overweight**	28 (22.6%)	27 (22.0%)		
**Obese**	92 (74.8%)	92 (74.8%)		

Pregnancy intention			χ^2^ = 36.54	<0.001
**Intended**	57 (46.0%)	102 (82.3%)		
**mistimed**	61 (49.2%)	18 (14.5%)		
**Unwanted**	6 (4.8%)	4 (3.2%)		

NB: IPI = interpregnancy interval, SD = standard deviation, CI = confidence interval, U = Mann–Whitney *U*-test.

**Table 3:** Comparison of the incidence of placenta previa and placenta accreta spectrum disorder between women with a normal and short interpregnancy interval.

Placenta Previa	Normal IPI (*n* = 124)	Short IPI (*n* = 124)	χ^2^	OR (95%CI)	*p*-value
Placenta previa by USS			2.41	2.32 (0.78–6.88)	0.13
**Present**	5 (4.0%)	11 (8.9%)			
**Absent**	119 (96.0%)	113 (91.1%)			

Placenta previa by inspection			2.16	2.11 (0.76–5.81)	0.15
**Present**	6 (4.8%)	12 (9.7%)			
**Absent**	118 (95.2%)	112(90.3%)			

Placenta accreta spectrum disorder at delivery			0.34	2.02 (0.18–22.13)	0.57
**Present**	1 (0.8%)	2 (1.6%)			
**Absent**	123 (99.2%)	122 (98.4%)			

NB: USS = ultrasound scan, IPI = interpregnancy interval.

**Table 4:** The association between sociodemographic factors and the presence of placenta previa using ultrasound among women with a short interpregnancy interval.

Variables	Placenta Previa		Test stat	df	*p*-value
	Present	Absent			
Mean age (SD)	35.09 (4.35)	33.00 (4.54)	*t* = −1.64 122		0.17

*Age group (years)*					0.11^*∗*^
**20-34 (*n*** **=** **75)**	4 (5.3%)	71 (94.7%)			
**>34 (*n*** **=** **49)**	7 (14.3%)	42 (85.7%)			

Marital status			χ^2^ = 2.99	2	0.86
**Married (*n*** **=** **121)**	11 (91.0%)	110 (90.9%)			
**Divorced (*n*** **=** **1)**	0 (0.0%)	1 (100.0%)			
**Widowed** (***n*** **=** **2)**	0 (0.0%)	2 (100.0%)			

Educational status			χ^2^ = 1.77	3	0.62
**No formal (*n*** **=** **2)**	0 (0.0%)	2 (100.0%)			
**Primary (*n*** **=** **4)**	1 (25.5%)	3 (75.5%)			
**Secondary (*n*** **=** **32)**	2 (6.3%)	30 (93.8%)			
**Tertiary (*n*** **=** **96)**	8 (9.3%)	78 (90.7%)			

Employment status					1.00^*∗*^
**Employed (*n*** **=** **109)**	10 (9.2%)	99 (90.8%)			
**Unemployed (*n*** **=** **15)**	1 (6.7%)	14 (93.3%)			

^*∗*^Fishers' exact test.

**Table 5:** The association between clinical factors and the presence of placenta previa using ultrasound among women with a short interpregnancy interval (*n* = 124).

Variables	Placenta previa by ultrasound		Test stat	*p* value
	Present	Absent		
Parity			*U* = 518.00	0.34
**Mean (SD)**	2.55 (1.44)	2.09 (0.94)		
**95% CI**	1.58–3.51	1.91–2.26		
**Median**	3.00	2.00		
**Min-Max**	1.00–5.00	1.00–4.00		

Number of C/S			*U* = 378.50	0.02
**Mean (SD)**	2.18 (0.08)	1.45 (0.65)		
**95% CI**	1.46–2.91	1.33–1.57		
**Median**	2.00	1.00		
**Min-Max**	1.00–4.00	1.00–4.00		

Mean gestational age **(**weeks)			*U* = 512.00	0.34
**Mean (SD)**	17.55 (6.02)	19.35 (5.76)		
**95% CI**	13.50–21.59	18.27–20.42		
**Median**	18.00	18.00		
**Min-Max**	7.00–28.00	9.00–32.00		

Mean systolic BP (mmHg)			*U* = 449.50	0.12
**Mean (SD)**	115.45 (16.35)	107.74 (11.32)		
**95% CI**	104.47–126.44	105.63–109.85		
**Median**	110.00	110.00		
**Min-Max**	90.00–140.00	80.00–140.00		

Mean diastolic BP (mmHg)			*U* = 579.00	0.69
**Mean (SD)**	67.73 (9.32)	67.26 (7.71)		
**95% CI**	81.47–73.99	65.82–68.69		
**Median**	70.00	70.00		
**Min-Max**	50.00–80.00	50.00–90.00		

Body mass index			χ^2^ = 0.56	0.91
**Underweight (n** **=** **1)**	0 (0.0%)	1 (0100.0%)		
**Normal (n** **=** **3)**	0 (0.0%)	3 (100.0%)		
**Overweight (n** **=** **27)**	3 (11.1%)	24 (88.9%)		
**Obese** (**n** **=** **92)**	8 (8.7%)	84 (91.3%)		

NB: C/S = cesarean section; IPI = interpregnancy interval, SD = standard deviation, CI = confidence interval, U = Mann–Whitney *U* test.

## Data Availability

The data used to support the findings of this study are available upon request from the corresponding author.
